# “Augmenting the referral pathway for retinal services among diabetic patients at Reiyukai Eiko Masunaga Eye Hospital, Nepal: a non-randomized, pre-post intervention study”

**DOI:** 10.1186/s12913-023-09105-3

**Published:** 2023-02-08

**Authors:** Ruchi Shrestha, Prerana Singh, Parami Dhakwa, Shailaja Tetali, Tripura Batchu, Pragati Shrestha Thapa, Varun Agiwal, Hira Pant

**Affiliations:** 1 Reiyukai Eiko Masunaga Eye Hospital, Banepa, Kavre Nepal; 2 SEVA Foundation, Kathmandu, Nepal; 3 Indian Institute of Public Health, Hyderabad, India

**Keywords:** Diabetes, Diabetic retinopathy, Screening, Health education

## Abstract

**Background:**

Diabetic Retinopathy (DR) is an important public health issue in Nepal. Despite the availability of retinal services, people may not access them because of the lack of knowledge about DR and poor referral systems. DR screening uptake was low at Reiyukai Eiko Masunaga Eye Hospital(REMEH) since retina services were started. Scheer Memorial Hospital is a multispeciality hospital near to REMEH. It has no eye department but has been running a regular diabetic clinic. This was a site for referring diabetic patients for DR screening. Improving DR awareness among general physicians has the potential to address these challenges.

**Methods:**

The aim of our study was to investigate the effectiveness of providing health education to selected health personnel and establish a referral pathway on the attendance of diabetic patients for retinal screening at REMEH. This was a non-randomized, pre-post intervention study design. Total of three health education sessions were provided to the health care professionals of Scheer on diabetic retinopathy using Power Point presentations, posters, pamphlets and videos. The study period was 16 months (2020 June –2021 September) and divided into 8 months pre-intervention(baseline data collection) and 8 months post intervention period. The proportional increase in number of diabetes attendance pre and post intervention was calculated by Z test. The change in knowledge of health care personnels pre and post intervention was scored and evaluated through a questionnaire and calculated by paired- t test. Data was analyzed using Excel and Epi Info 7.The Protocol was published on August 21, 2021, in JMIR Publications.

**Results:**

The proportional increase in number of referrals of diabetes attendance post intervention increased from 50 to 95% and was statistically significant (*p* < 0.001, 95% CI: 0.214–0.688). The mean score of knowledge gained by physicians on DR awareness was more at post intervention (8.8 ± 1.32) than pre intervention (6.4 ± 1.51). It was statistically significant (*p* < 0.001).

**Conclusion:**

This study shows that a well-planned health education intervention changes the knowledge in physicians about DR. There is an increase in the number of referrals and attendance of patients for DR screening with the change in knowledge and referral mechanism.

**Trial Registration:**

Clinical Trials.gov NCT04829084; https://clinicaltrials.gov/ct2/show/NCT04829084:02/04/2021.

**Supplementary Information:**

The online version contains supplementary material available at 10.1186/s12913-023-09105-3.

## Background

Diabetic retinopathy (DR) is a complication of diabetes damaging the retinal vessels that can lead to blindness if left untreated [1]. More than 75% of people with diabetes for 20 years or more have some form of DR and 10% have retinopathy requiring treatment. Timely screening, early detection and treatment can reduce the risk of blindness by more than 90% [2].

The worldwide prevalence of Diabetic Retinopathy (DR) was found to be 34.6% [3]. It is the fifth leading cause of visual impairment and the fourth leading cause of blindness in the world. DR is responsible for 4.8% of the 37 million cases of blindness throughout the world [4].

DR is an emerging cause of blindness in developing countries like Nepal. SK Mishra et al. showed that 10% of the people with diabetes had some form of DR in Nepal. The prevalence of Non-proliferative Diabetic Retinopathy, Proliferative Diabetic Retinopathy, and complete vision loss due to macular edema was found to be 9.1%, 0.5%, and 0.3% respectively in Nepal [5]. The awareness of retinopathy is very poor in Nepal and the public should be sensitized about diabetic eye diseases [6].Timely referral of Diabetes Mellitus(DM) patients to retina centers for screening is likely to have an early diagnosis of DR [7].

Our organization, Reiyukai Eiko Masunaga Eye Hospital (REMEH) is a non-profitable community-based hospital in Banepa, Nepal providing eye care services to a population of 411,057 in the Kavrepalanchowk district. In 2019, the hospital launched the retina clinic and started retinal services. Since the uptake of DR screening did not increase as expected, we conducted a problem tree analysis and identified that lack of referral system is one of the major reasons for the low uptake of DR screening at our hospital. Scheer Memorial Hospital is a multispeciality hospital about 2 km distance to Reiyukai Eiko Masunaga Eye Hospital. It has no eye department but has been running a regular diabetic clinic. This is a site for referring diabetic patients for DR screening. We identified that creating referral pathway between Scheer and REMEH would increase the uptake of diabetic retinopathy. Similarly, Piyasena et al. identified that the lack of knowledge and awareness on DR, and zero awareness of the importance of regular DR screening and follow-up, combined with poor information on referral pathways were key elements to be improved for better uptake of DR in the context of Sri Lanka [8].

Published studies on referral pathways in Nepal are scarce. The aim was to investigate the effectiveness/ impact of providing health education intervention to selected health personnel and establishing a referral pathway on the attendance of patients with DM for retinal screening at REMEH. This was a pilot study as no such study was previously conducted in Nepal.

## Method

### Research Objective

The aim of this study was to increase retinal screening uptake among patients with DM and to decrease DR-associated blindness by augmenting the referral pathway in a selected hospital in Nepal.

### Hypothesis

Providing DR health education to selected health personnel and creating a referral pathway would increase the uptake of retinal services (screening and treatment) by diabetes patients referred to REMEH.

### Study design

This was a non-randomized pre- and post-intervention study without a control group.

### Study setting

Reiyukai Eiko Masunaga Eye Hospital (REMEH) is an eye hospital. Scheer Memorial Hospital is the intervention hospital. It is a multispeciality hospital with no eye department and has been conducting regular diabetic clinics.There was no baseline data on referral of patients from Scheer to REMEH before the study.

### Study period

The total duration was 16 months from June 2020 to September 2021. The initial 8 months from June 2020 to January 2021 was for base line data collection (pre-intervention period). The remaining 8 months from February 2021 to September 2021 was the post intervention period.

### Study participants

Selected Health personnel of Scheer Memorial Hospital (The intervention hospital) who are directly involved in providing health services to diabetes mellitus patients in the diabetic clinic at Scheer. Physicians, paediatricians, medical officers and their assistants were included. Those health personnel who didn't attend at least 2 sessions of intervention were excluded.

List of health personnel

**Table:** 

Physicians	1. Internal medicine	3
	2. Paediatricians	3
	3. Medical officer	4
Non-Physicians	1. Paediatric outpatient department Assistant	1
	2. Medical Assistant	1
	3. Medicine Assistant	2

### Sampling techniques

Complete enumerations of all health personnel managing patients with diabetes in the intervention hospital.

### Inclusion criteria

All health personnel involved in the management of patients with diabetes at the intervention hospital.

### Materials

The Information Education and Communication (IEC)material developed by the Indian Institute of Public Health-Hyderabad; India (IPHH) was used for intervention after converting into Nepalese language. The IEC materials were PPT, poster (Annex 1), pamphlets (Annex 2, 3) and video. Referral slips (Annex 4) were also used. The PPT, posters and pamphlets contained introduction of diabetic retinopathy, its etiology, types of diabetic retinopathy and management. The video showed the etiology of diabetic retinopathy, its symptoms, signs and management. All IEC materials explained the importance of timely referral on prevention of blindness. The referral slip contained the name of both hospitals for two-way communication. Change in knowledge of health personnel was assessed by pre- and post-assessment(intervention) questionnaire prepared using Public Health Foundation of India (PHFI)’s “Certificate Course in Evidence Based Management of Diabetic Retinopathy (CCDR) assessment questionnaire” as a guide (Annex 5). The first five questions were on diabetes and complications and the remaining five questions were specific to DR. A score of one was given for each question answered by the health professional. The total score was 10/10 if all the questions were correctly answered. Mean score is the score gained by the health personnel from the pre and post assessment questionnaire.

### Implementation of intervention

The retina specialist and the optometrist/outreach coordinator of REMEH conducted the intervention. The assistant manager of the hospital was responsible for the logistics. There was no control group in this study. A pilot was done before the main intervention started.

A pilot was conducted In Feb 19,2021 to the health professionals of Krishna Prasad Hospital on Feb1^st^ 2021 before the main intervention. All materials were explained and shown. Pre and post-test evaluation was also done through the questionnaire. One participant scored 4 out of 10 before intervention 9 post intervention and the second participant scored 7 & 9 before and after intervention respectively. There was improvement in the scoring of health personnel. Pilot helped us improve our shortcomings in the main intervention.

The intervention for selected health personnel of Scheer was conducted after 8 months of baseline data collection as a once-a-month health education session for three months. (February 2021-April 2021) in REMEH. The post intervention data collection started from February 2021 as soon as the first intervention started.

First intervention was conducted on health personnel of Scheer memorial Hospital on February 6, 2021. Proper COVID-19 guidelines were followed with social distancing and masks. The participants were provided with transportation and refreshments.

Before we began our intervention, all the participants signed the consent form that included details of age, gender, qualification, and years of experience. In the first session, we conducted a pre- intervention assessment by providing a baseline questionnaire to the participants. Questionnaire was translated into native language. At the end we judged their knowledge with pre intervention questionnaire. We made sure all questions were attempted. The principal investigator, retina specialist made power point presentation.

We conducted the second round of intervention on March 16, 2021.Fourteen participants attended the session. Two medical officers from the previous session did not participate and two new medical officers participated in the second session. Our optometrist distributed the pamphlets and explained the contents. Participants were reminded to counsel DM patients to visit REMEH, use referral slip while referring patients to REMEH, handover DR-related pamphlets to DM patients and finally record total cases referred to REMEH in a month.

The third and final intervention was held on April 23, 2021. Total of 14 participants participated in this session. The session started with post intervention questionnaire. We collected answer sheets and gave answers to all questions in an interactive session. At the end we distributed additional referral slips and pamphlets.

The same questionnaire was shared with all the participants via goggle form after 2 months on July1, 2021. All the 14 participants filled the answers.

### Data collection

Data collection included the pre-post intervention after health education at Scheer Memorial, as well as patient referral data at REMEH. Data was entered into the EXCEL sheet every week. The data tools are mentioned in the Annex (1–5).

### Data analysis

The baseline and post intervention patient referral data was compared and the proportional increase due to intervention was calculated. The change in knowledge of health personnel was assessed by pre and post assessment questionnaire. In patients’ data, Information on DR and duration of DM was collected, visual acuity and stage of DR was noted. Descriptive analysis for the same was done. Information on the referred data was collected from the intervention hospital. Mean median and Standard deviation were calculated for the demographic variables. Z test was applied to determine the number of referrals to justify the research hypothesis that the proportion of referral cases after the intervention is improved. The change in mean knowledge after the intervention, i.e. the mean knowledge score pre and post-intervention are tested using the paired t-test.Data was analyzed using excel and Epi info 7. The ethical approval for this study was obtained from the Ethical Review Board of Nepal Health Research Council on 22/12/2020(ERB Protocol Registration Number#582/2020P).

Protocol of the study was published on August 21, 2021, in JMIR Publications. Clinical trials registration: NCT04829084 [9].

### Outcome

Primary outcome:

- 1. Change in the proportion of referred patients from Scheer Memorial to REMEH when compared to baseline referrals before the intervention.

Secondary outcome:

- 2.Change in knowledge on DR in the Health Care Personnel (HCP) who participated in health education sessions in Scheer Memorial.

## Results

Of the 14 health care personnel enrolled in health education intervention 10(71.43%) were physicians with median experience of 4.5 years (IQR 3–6.75) and 4 (28.57%) were non physicians with median experience of 27.5 years. The male and female ratio in health care personnel was equal with an mean age of 35.8 years (SD ± 9.4) years (Table 1).

**Table 1:** Socio demographic profile of the Health care providers

Variables	Categories	N (%)	Age (in years)		
			Mean ± SD	Min	Max
Sex	Male	7(50)	34.57 ± 7.74	27	51
	Female	7(50)	37 ± 10.64	24	57
Mean Age, years		14 (100)	35.79 ± 9.38	24	57
Qualification	Physician	10(71.4)	32.1 ± 3.56	27	39
	Non-physician	4(29.6)	45 ± 12.55	24	57
Experience, years: Median (IQR)		14 (100)	4.5 (3–6.75)	1	38

The mean score of the physicians DR knowledge and awareness increased at post intervention (8.8 ± 1.32) as compared to pre intervention (6.4 ± 1.51) (*p* < 0.001) whereas for non-physicians’ group pre and post score was 5 ± 1.5 and 6.25 ± 1.26 with *p* = 0.3416. Overall mean score at pre and post intervention was 6 ± 1.52 and 8.07 ± 1.73 respectively (Table 2). The mean score of the physicians DR knowledge and awareness remained same (8.8 ± 0.92) as compared to post intervention. Mean score of non-physicians DR knowledge and awareness decreased (5.00 ± 2.58). Overall mean score at follow up was 7.71 ± 2.30.

**Table 2:** Change in knowledge level of health care personnel at pre and post intervention

Type of health professionals	Pre intervention score (Mean ± SD)	Post intervention score (Mean ± SD)	*P*-value (paired t-test)	95% CI
Physician	6.4 ± 1.51	8.8 ± 1.32	< 0.001	(1.27, 3.52)
Non-physician	5 ± 1.15	6.25 ± 1.26	0.3416	(-7.01,9.30)
Overall	6 ± 1.52	8.07 ± 1.73	< 0.001	(0.77, 3.37)

**Table 3:** Proportion of referral to REMEH from Scheer in per and post intervention period

Intervention	No. of patients who presented to REMEH	Total Cases referred by HP	Attendance out of referral %	z-test	*p*-value	95% CI
Pre	9	18	50.00	4.85	< 0.0001	(0.217,0.691)
Post	62	65	95.38			

Total of 71 patients with Diabetes Mellitus attended from intervention hospital (Scheer) during the study period from 1^st^ June 2020 to 30^th^ September 2021(16 months). During the baseline data collection period (preintervention period) of 8 months, total of 9 patients attended REMEH. Scheer had referred 18 patients in the baseline data collection period of 8 months. Sixty-two patients from Scheer attended REMEH after the intervention. Scheer had referred 65 patients in the post intervention period of 8 months. So pre intervention attendance among referral was 50% and post intervention attendance was 95%. The proportional increase in the number of referrals after the intervention was statistically significant (*p* < 0.001, 95% CI; 0.214–0.688) (Table 3).

Referral cases from Scheer were further divided based on the experience and position of health care physicians. Total of 8 (80%) health care personnel had experience of less than or equal to 5 years and they referred a total of 8 (88.89%) and 44 (70.97%) patients from pre and post intervention period and rest of the patients were referred by physicians with > 5 years’ experience. Internal medicine doctors referred more, followed by medical officer and pediatricians but it was not statistically significant (Table 4).

**Table 4:** Health care personnel experience and post associated with Referral From Scheer

Variable	Categories	No. of Doctors	Total cases attended	Cases referred from Scheer (%)		*p*-value
				Pre intervention (%)	Post intervention (%)	
Experience	< = 5	8	52	8(88.89)	44(70.97)	0.4279
	> 5	2	19	1(11.11)	18(29.03)	
Position/ Post	Pediatric	3	5	2(22.2)	3(4.8)	0.0688
	Medical Officer	4	18	3(33.3)	15(24.1)	
	Internal Medicine	3	48	4(44.5)	44(70.9)	

In Pre-intervention period, 6 (66.67%) males and 3 (33.33%) females attended REMEH, whereas 24(38.71%) males and 38(61.29%) females attended REMEH at post intervention. The average age of pre intervention patients was 56.67 years (SD ± 17.30) while that of post intervention patients was 55.39 years (SD ± 12.51). Mean duration of diabetes in the pre intervention was 7.05 (SD ± 6.37) and post intervention was 5.12 (SD ± 5.80). Almost 66.67% patients had smoking habit in per intervention whereas post intervention was 48.39% patients. A total of 7 (77.78%) and 45 (72.78%) patients were a normal lipid profile. It was observed that basic and clinical characteristics were not significantly different in pre and post intervention group (Table 5). Chi-square test was applied for the qualitative data and unpaired t- test was applied for the quantitative data to calculate the *p*-value.

**Table 5:** Socio demographic and clinical profile of the patients attended at REMEH

Variable	Categories	Pre intervention (%)	Post intervention (%)	*p*-value
Gender	Male	6 (66.67)	24(38.71)	0.113
	Female	3 (33.33)	38(61.29)	
Age, years (Mean ± SD)		56.67 ± 17.30	55.39 ± 12.51	0.786
Duration of diabetics (Mean ± SD)		7.05 ± 6.37	5.12 ± 5.80	0.361
Drug administration	Oral	1(11.11)	31(50.00)	0.081
	Insulin	6 (66.67)	21(33.87)	
	Oral and Insulin	2(22.22)	10(16.13)	
History of smoking	Smoking	3(33.33)	11 (17.74)	0.272
	Not smoking	6(66.67)	51 (82.26)	
Diet	Control	8 (88.89)	50 (80.65)	0.550
	Not control	1 (11.11)	12 (19.35)	
Lipid profile	Abnormal	0 (0)	6 (9.68)	0.329
	Normal	9 (100)	56 (90.32)	
Intra ocular pressure	Left eye	16.78 ± 6.06	17.05 ± 5.05	0.884
	Right eye	16.89 ± 4.01	16.00 ± 4.18	0.551

Table 5 shows the socio-demographic and clinical profile of the patients who attended REMEH.

## Discussion

We conducted a non-randomized pre-post health education intervention study on health personnel with an objective to increase the referral of DM patients for DR screening. The health professionals were provided health education to create awareness and knowledge of DR using PPTs, posters, pamphlets, and referral slips. There was a significant increase in referral and attendance of DM patients in REMEH for DR screening after the intervention. There was also a significant increase in knowledge among the physicians after the intervention.

Few studies in Nepal have mentioned the need of stakeholders to enhance and provide quality eye care services to diabetic people. The need to establish coordination between general medical service providers and the eye care service providers to develop a coordinated service system for diabetes and diabetic retinopathy care has also been discussed [5–7]. There are no studies done involving stakeholders in health education intervention sessions to improve the uptake of screening in Nepal till date.

Our study should be the first study involving stakeholders in health education session on DR aiming to increase the DR referral in Nepal. Piyasena et al. in their study involved patients with diabetes mellitus and stakeholders in health education intervention sessions to improve the uptake of screening for DR. They also developed strategies to improve knowledge on diabetic retinopathy in Sri Lanka, like our study [9, 10].

The male and female ratio in health care personnel was equal with overall average age of 35.8 years Ten (71.43%) were physicians with median experience of4.5 years and 4 (28.57%) were non physicians with median experience of 27.5 years. Anwar et al. in their study in Pakistan enrolled 36 physicians for DR related health education intervention. The mean age was33.10 ± 11.2 years and 64% were female and 36% were male. Most of the participating physicians, 61% (*n* = 22) had spent less than 5 years in practice [11].

In our study, the mean score of the physicians DR knowledge and awareness increased at post intervention as compared to pre intervention. We did a follow up after 2 months by sending questionnaire via goggle forms. All the participants except the helpers responded and the overall mean score was 7.71 ± 2.30 not decreased for the physicians. Similarly other studies have also assessed the DR awareness of physicians by calculating Diabetic retinopathy awareness index (DRAI) and found the need of referral communication with the physicians and ophthalmologists/retina specialist [11, 12].

Similarly, we used a referral slip as a mode of communication between an ophthalmologist and health care personnel providing medical care to the patients with diabetes at Scheer Memorial hospital. Storey et al. also found that written communication between an ophthalmologist and a primary care physician (PCP) and referral vice versa was effective to change the behavior of the referring physicians [13]. In our study, we also observed that a two-way communication using referral slip was very effective in increasing the referrals of diabetic retinopathy.

We also conducted a pilot before the health education intervention and there were only two physicians. Therefore, it was easy to compare the before and after score of the physicians. Pilot made us realize that pre post questionnaire needed to be written in Nepali language as well, to eliminate language barrier. We missed to distribute information sheet and consent form which we corrected during the final sessions. We used posters, pamphlets, videos and PPTs with diabetic retinopathy awareness message in our educational sessions which helped change the behavior of the physicians. Similarly, printed educational messages and posters increased behavior and change in attitude of primary care physicians and assistants of a multi-specialty hospital in Ireland and increased diabetic retinopathy screening like our study [14, 15]. But we did not find any studies that conducted pilot to improve the missing factors to conduct an effective intervention.

There was a significant increase in the number of diabetic retinopathy patients attendance post intervention in our study. The use of printed educational messages like the use of pamphlets and referral slips also increased the number of referrals by primary health care professionals to ophthalmologist [16–18]. There was a monthly variation with an increase and decrease in the number of DM patients for DR screening during the post intervention due to COVID but finally there was a significant increase in the number of referrals.

Few studies have shown that lesser experienced primary health care professionals and internists could miss the diagnosis and refer a smaller number of diabetic retinopathy than the experienced health care professionals [12, 19]. Similarly, in our study internal medicine doctors referred more, followed by medical officer and pediatricians. The relationship between years of experience and referral was not statistically significant in our study, and we need to conduct studies with larger sample size in future.

In our study female patients attended for diabetic retinopathy screening more than males in both the pre and post intervention period. Similarly in other study females were found to be more than males for diabetic retinopathy screening [13]. This could also be addressed in future studies.

The mean age of pre intervention patients was 56.67 years (SD ± 17.30) while that of post intervention patients was 55.39 years (SD ± 12.51). Similarly in few other studies the mean age 55.43 ± 11.86 years [5, 6]. Mean duration of diabetes of the patients that attended for diabetic retinopathy study in the pre intervention was 7.05 (SD ± 6.37) and post intervention was 5.12 (SD ± 5.80). It was observed that basic and clinical characteristics were not significantly different in pre and post intervention group. Our study was more focused on the referral system and change in knowledge of the health care professionals so further studies are required to be focused on the clinical outcome of diabetic patients after health education sessions.

## Conclusion

A well-planned health education intervention changes the knowledge in physicians about DR. With the change in knowledge and structured referral system, there is significant increase in the number of referrals and attendance of patients for DR screening. This study clearly shows the need to mobilize and link physicians in general hospital with Eye Hospital.

### Strength and limitations

The strength of our study is that it is one of the first studies in Nepal that includes health care professionals involved in DM management and strengthens the referral for the screening of diabetic retinopathy. The pilot gave us information on re-comprehension of the content of the materials to be used for the main study.

Due to COVID-19, the number of selected health personnel participating in health education was less than expected. We did not have a control hospital to compare our intervention and the effect of the referral pathway in 2 hospitals, which could limit our study’s generalizability. Due to COVID-19 the number of patients attending Scheer and REMEH might have been affected. It will be useful to have further studies with a qualitative component to understand the barriers faced by health personnel on the referral process and reasons for delays, if any.

## Supplementary Information

**Additional file 1: Annex 1. **Poster on Diabetic Retinopathy. **Annex 2 **and** 3.** Pamphlet on Diabetic Retinopathy.** Annex 4.** Referral Slip.** Annex 5.** Pre and post evaluation questionnaire.

## Data Availability

Datasets generated during and/or analyzed during the current study are available from the corresponding author on reasonable request.

## Abbreviations

DM: Diabetes mellitus
DR: Diabetic retinopathy
IEC: Information education and communication
REMEH: Reiyukai Eiko Masunaga Eye Hospital
