# Supplementary material for: “Augmenting the referral pathway for retinal services among diabetic patients at Reiyukai Eiko Masunaga Eye Hospital, Nepal: a non-randomized, pre-post intervention study”
Source: BMC Health Serv Res. 2023 Feb 8;23:126. doi: 10.1186/s12913-023-09105-3 (PMC9905012; doi:10.1186/s12913-023-09105-3)
Supplement: Supplementary file 1 — Additional file 1: Annex 1. Poster on Diabetic Retinopathy. Annex 2 and 3. Pamphlet on Diabetic Retinopathy. Annex 4. Referral Slip. Annex 5. Pre and post evaluation questionnaire. [file 12913_2023_9105_MOESM1_ESM.pdf]

Annex 1:

Poster on Diabetic Retinopathy

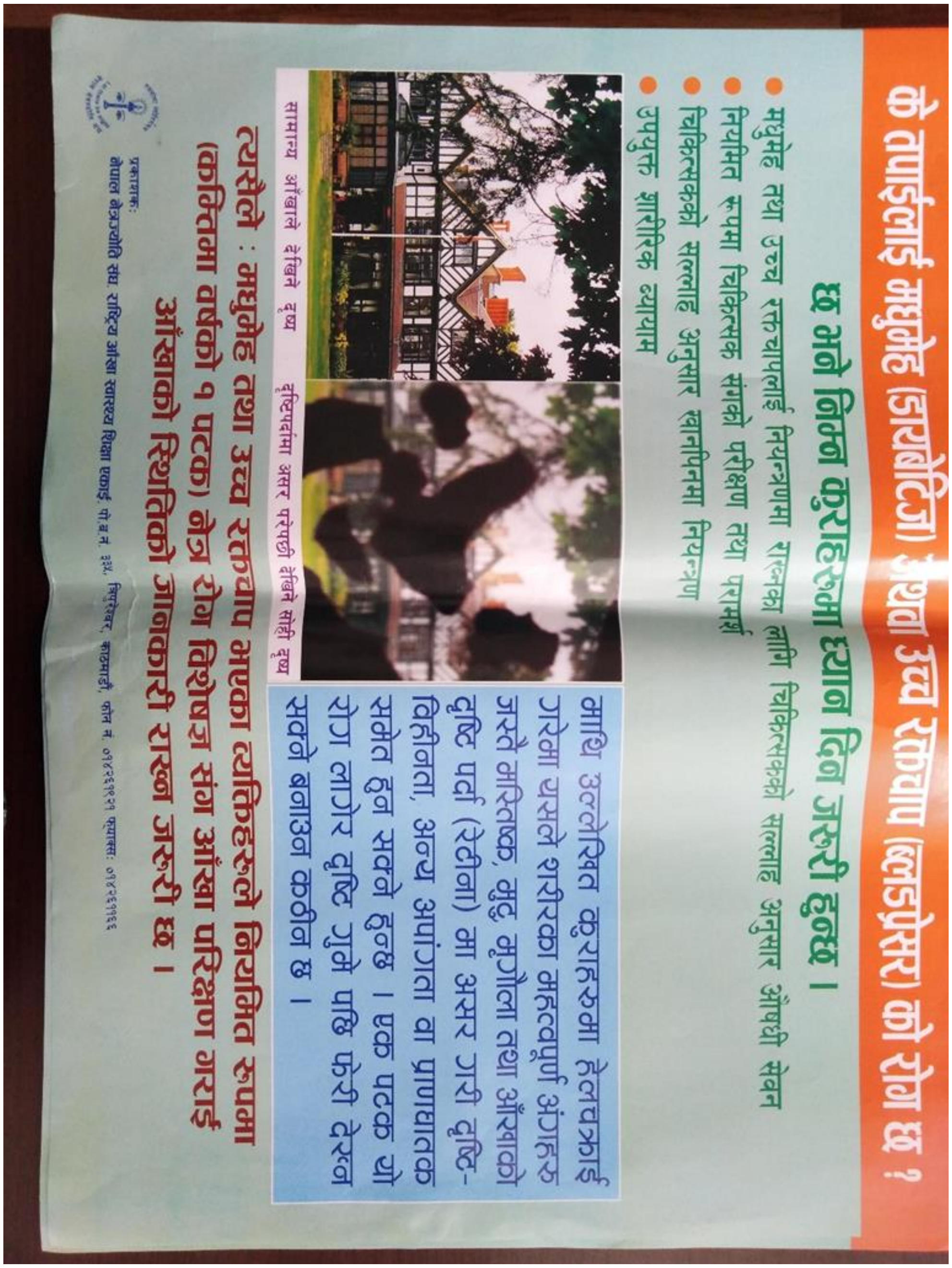

Annex 2 and 3:

Pamphlet on Diabetic Retinopathy

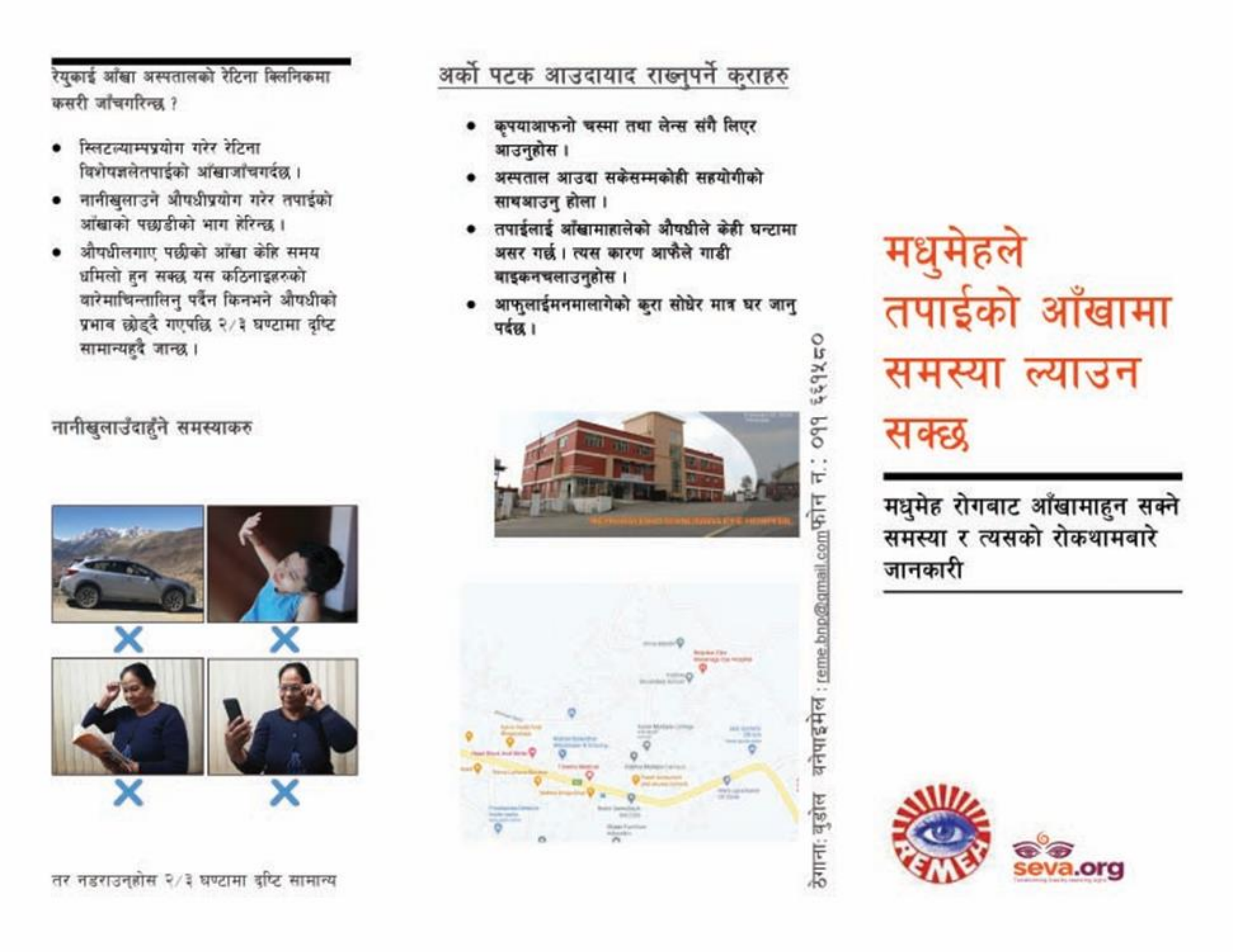

रेयुकाई आँखा अस्पतालको रेटिना क्लिनिकमा कसरी जाँचगरिन्छ ?

- स्लिटल्याम्पप्रयोग गरेर रेटिना विशेषज्ञलेतपाईको आँखाजाँचगर्दछ ।
- नानीखुलाउने औषधीप्रयोग गरेर तपाईको आँखाको पछाडीको भाग हेरिन्छ ।
- औषधीलगाए पछीको आँखा केहि समय धमिलो हुन सक्छ यस कठिनाइहरुको बारेमाचिन्तालिनु पर्दैन किनभने औषधीको प्रभाव छोड्दै गएपछि २/३ घण्टामा दृष्टि सामान्यहुदै जान्छ ।

नानीखुलाउँदाहुँने समस्याकरु

तर नडराउनुहोस २/३ घण्टामा दृष्टि सामान्य

अर्को पटक आउदायाद राख्नुपर्ने कुराहरु

- कृपयाआफनो चस्मा तथा लेन्स संगै लिएर आउनुहोस ।
- अस्पताल आउदा सकेसम्मकोही सहयोगीको साथआउनु होला ।
- तपाईलाई आँखामाहालेको औषधीले केही घन्टामा असर गर्छ । त्यस कारण आफैले गाडी बाइकनचलाउनुहोस ।
- आफुलाईमनमालागेको कुरा सोधेर मात्र घर जानु पर्दछ ।

ठेगाना: थुडोल यनेपाइमेल : reme.bnp@gmail.comफोन न.: ०११ ६६१५८०

मधुमेहले तपाईको आँखामा समस्या ल्याउन सक्छ

मधुमेह रोगबाट आँखामाहुन सक्ने समस्या र त्यसको रोकथामबारे जानकारी

REMEH

seva.org

मधुमेहलेआँखामालाग्न सक्ने रोग रेटिनोप्याथी, एक चिनारी

मधुमेह रेटिनोप्याथीविकाशोन्मुख देशहरुमाअन्धोपनको उदयीमानकारण हो। W.H.O को अनुमान छ कीD.Rसंसार भरिनै अन्धोपनको ३७ करोडमध्य ४८%को लागीजिम्मेवार छ। २० वर्षभन्दामाथिकामानिसहरुमा नेपालमामधुमेहको प्रकोप १४. ६ % र ४० वर्षमाथिकामानिसहरुमा १९ %भन्दा बढी रहेको छ।लगभग १०% मधुमेह रोगीहरु नेपालमा रेटिनोप्याथीभएको पाइएको छ।

रेयुकाई आँखा अस्पताल पुगेपछि के गर्ने त ?

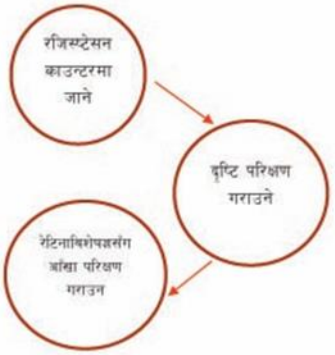

<u>मधुमेहरोगीकालागीप्रारम्भिक र नियमितआँखाजाँचकिन महत्वपूर्ण छ ?</u>

- मुख्यविशेषता के छ भने तपाइले पहिलो चरणमा कुनै पनिआँखा रोग सम्बन्धित लक्षण देख्नुहुन्न।
- प्रारम्भिकआँखाजाँचले आफु सजगहुनआँखाको पर्दा पछाडि आएको परिवर्तन देखाउछ।
- यदिआँखामाआएको परिवर्तन समयमापत्तालाग्यो भने आँखामाउपचार प्रभावकारी रहन्छ र दृष्टिहीन हुन बाट जोगाउछ।
- त्यसकारण नियमीतआँखाजाँचआवश्यक छ।

के हामी यस अवस्थाबाट बच्नमोतिविन्द् शल्यक्रियागरि अथवा पढ्न र टाढाको वस्तु हेर्न चस्माको प्रयोग गर्न सक्छौं

- मधुमेहबाट आँखामाभएको परिवर्तनहरु मोतिविन्दु वा चस्माका साथभ्रमितगर्नुहुदैन।
- हामीआँखाको बाहिरी भागको त्रुटिहरु सुल्फाउनवा लेन्स पावरका परिवर्तनको सच्याउनचश्माप्रयोग गर्छौ।
- जबप्राकृतिक लेन्स अपारदर्शी र बाक्लो हुन्छ तबमोतिविन्दु शल्यक्रिया र intra ocular lens प्रत्यारोपण गरिन्छ।
- त्यसकारण चस्माको प्रयोग वामोतिविन्दुको शल्यक्रिले मधुमेहले गर्न सक्ने क्षति रोक्न सक्दैन।
- यस बाहेककुनै पनिआँखाको ड्प्स वा स्थानियउपचारले यस अवस्थालाई रोक्दैन।
- त्यसकारण तपाइले मोतिविन्दुको शल्यक्रिया गरेपछि पनिअथवाचश्माप्रयोग गर्दै हुनुहुन्छ भने पनिमधुमेहका विरामीलेआँखाजाँचगर्नुपर्नेछ

Annex 4:

Referral Slip:

रेयूकाई एईको मासुनागा आँखा अस्पताल

डाइबेटिक रेटिना आँखा जाच

रिफरल स्लिप

नाम :	मिति:
Name	Date
ठेगना :	फोन नं :
Address:	Phone no:
उमेर :	लिङ्ग
Age :	Sex

रिफर डाक्टर ____________________

Referral Doctor: ____________________

नोट ____________________

Note ____________________

रिफर अस्पताल : शीर मेमोरियल अस्पताल ,बनेपा

Referral Hospital :Scheer Memorial Hospital Banepa

Annex 5:

Pre and post evaluation questionnaire:

Diabetes Eye Care Education Initiative

Baseline Questionnaire

PLEASE TICK / CIRCLE THE ANSWERS

A. **Out of all the Outpatients/Inpatients seen by you, what percentage of people with diabetes you see in a week?**

a) 2% - 5%

b) 5% - 10%

c) 10% - 20%

d) More than 20%

B. **Out of total number of people with diabetes, what percentage constitutes people with Diabetic Retinopathy?**

(a) < 5%

(b) 5% - 10%

(c) 10% - 20%

(d) More than 20%

---

1. **One of the most common causes of blindness in the working population.**

   a. Cataract

   b. Untreated DR

   c. Refractive Error

d. None

2. Retinal examination must be done in all people with diabetes, irrespective of the type of diabetes

a. Once in 3 years

b. Once in 5 years

c. Once a year

d. When the patient has vision complaints

3. The symptoms produced in diabetic retinopathy are

a. Watering

b. Itching

c. Redness and discharge

d. Blurred vision

4. The pathogenesis of diabetic retinopathy is due to

a. Microvascular occlusion

b. Microvascular leakage

c. Both of the above

d. None of the above

5. The most important non-modifiable risk factor associated with DR is

a. Anemia

b. Cardiovascular disease

c. Duration of diabetes

d. Smoking

6. All patients with diabetic retinopathy require

a. Laser photocoagulation

b. Glycemic control

c. Intravitreal anti-VEGFs (vascular endothelial growth factor)

d. Ocular surgery

7. Which of the following is the first sign of diabetic retinopathy?

a. Hard-exudates

b. Cotton wool spot

c. Micro aneurysm

d. Flame Shaped haemorrhages

8. Diabetic macular edema is treated with

a. Laser

b. Anti-VEGF

c. Both of the above

d. None of the above.

9. Which of following is an indication for panretinal laser photocoagulation

a. Moderate non-proliferative diabetic retinopathy

b. Proliferative diabetic retinopathy

c. Mild non-proliferative diabetic retinopathy

d. Diabetic macular edema

10. Which of the following is NOT an indication for vitrectomy

a. Non-clearing vitreous haemorrhage

b. Tractional retinal detachment

c. Proliferative diabetic retinopathy

d. Diabetic macular edema

Diabetes Eye Care Education Initiative

Post Training Questionnaire

PLEASE TICK / CIRCLE THE ANSWERS

1. **One of the most common causes of blindness in the working population.**
   a. Cataract
   b. Untreated DR
   c. Refractive Error

2. **Retinal examination must be done in all people with diabetes, irrespective of the type of diabetes**
   e. Once in 3 years
   f. Once in 5 years
   g. Once a year
   h. When the patient has vision complaints

3. **The symptoms produced in diabetic retinopathy are**
   e. Watering
   f. Itching

g. Redness and discharge

h. Diminution of vision

4. The pathogenesis of diabetic retinopathy is due to

e. Microvascular occlusion

f. Microvascular leakage

g. Both of the above

h. None of the above

5. The most important non-modifiable risk factor associated with DR is

e. Anemia

f. Cardiovascular disease

g. Duration of diabetes

h. Smoking

6. Which of the following is the first sign of diabetic retinopathy?

a. Hard-exudates

b. Cotton wool spot

e. Micro aneurysm

f. Flame Shaped haemorrhages

7. All patients with diabetic retinopathy require

a. Laser photocoagulation

b. Glycemic control

c. Intravitreal anti-VEGFs (vascular endothelial growth factor)

d. Ocular surgery

8. Diabetic macular edema is treated with

a. Laser

b. Anti-VEGF

c. Both of the above

d. None of the above.

9. Which of following is an indication for panretinal laser photocoagulation

a. Moderate non-proliferative diabetic retinopathy

b. Proliferative diabetic retinopathy

c. Mild non-proliferative diabetic retinopathy

d. Diabetic macular edema

10. Which of the following is NOT an indication for vitrectomy

a. Non-clearing vitreous haemorrhage

b. Tractional retinal detachment

c. Advanced Proliferative diabetic retinopathy

d. Diabetic macular edema

Thank You for your Participation in the survey!

Additional file 6:

Patient consent form:

Informed Consent form

Study Title: Augmenting the referral pathway to improve the uptake of retinal services among Diabetic patients at Reiyukai Eiko Masunaga Eye Hospital, Nepal.

Name: ____________________

Date of Birth/Age: _________________

Why is this study being done?

We are doing this study to save vision of patients with Diabetes. Diabetic retinopathy is a common and specific microvascular complication of diabetes, and remains the leading cause of preventable blindness in working-aged people. . Diabetic Retinopathy if detected early can be treated to preserve the vision. Once vision has been lost it cannot usually be restored.

Please do initial in box (Subject)

(i) I confirm that I have read information sheet provided and explained about the study [ ]

(ii) I understand that the researchers and the IRB and other regulatory authorities will not need my permission for further research that may be conducted in relation to it, even if I withdraw from the trial. I agree to this access. However, I understand that my identity will not be revealed in any information released to third parties or published. [ ]

(iii) I agree not to restrict the use of any data or results that arise from this study provided such a use is only for scientific purpose(s) and I agree to take part in the above study. [ ] [ ]

Signature (or Thumb impression) of the Subject/Legal Guardian: ____________

Date: _____/_____/______ Signatory's

सु -सूचित मन्जुरीनामा

अध्ययन शीर्षक: Augmenting the referral pathway to improve the uptake of retinal services among Diabetic patients at Reiyukai Eiko Masunaga Eye Hospital, Nepal

नाम :

जन्म मिति ।उमेर :

म ....उमेर ....वर्ष ले यस संस्थानबाट गर्न लाग्नु भएको माथि उल्लेखित _शिर्षकको अनुसन्धान सम्बन्धी 'जानकारी पत्र र प्रश्नोत्तर समेत गरेर यो अध्ययन-अनुसन्धान सम्बन्धमा जानकारी प्राप्त भयो।

यो अनुसन्धानकार्यमा मेरो सहभागिता मेरो व्यक्तिगत इच्छामा भर पर्ने भन्ने कुरा मैले बुझेको छु। यसको लागि मैले कुनै कारण दिनु नपर्ने र त्यसबाट मैले पाउने सेवा र मेरो कानुनी अधिकारमा असर नपर्ने समेत मलाई बुझाईएको छ।

यस अनुसन्धानको प्रतिवेदन वा सम्बन्धित प्रकाशित कृतिहरुमा मेरो कुनै व्यक्तिगत परिचय खुल्ने जानकारी प्रकाशित हुने छैन भन्ने कुरा मैले बुझेको छु।

यी सबै कुराहरु जानी/बुझी, म यस अध्ययन अनुसन्धानमा सहभागी हुन स्वेच्छाले राजी भई यो सुसूचित मन्जुरीनामामा सहिछाप गरेको छु।

सहभागीको सही:-

सहभागीको नाम, थर :

मिति :

सहभागीको बुढीऔंलाको त्याप्चे

दायाँ	बायाँ

मिति :-
